# Supplementary material for: A signature-agnostic test for differences between tumor mutation spectra reveals carcinogen and ancestry effects
Source: Commun Biol. 2026 Feb 20;9:462. doi: 10.1038/s42003-026-09652-5 (PMC13035805; doi:10.1038/s42003-026-09652-5)
Supplement: Supplementary file 1 — Supplementary Information [file 42003_2026_9652_MOESM1_ESM.pdf]

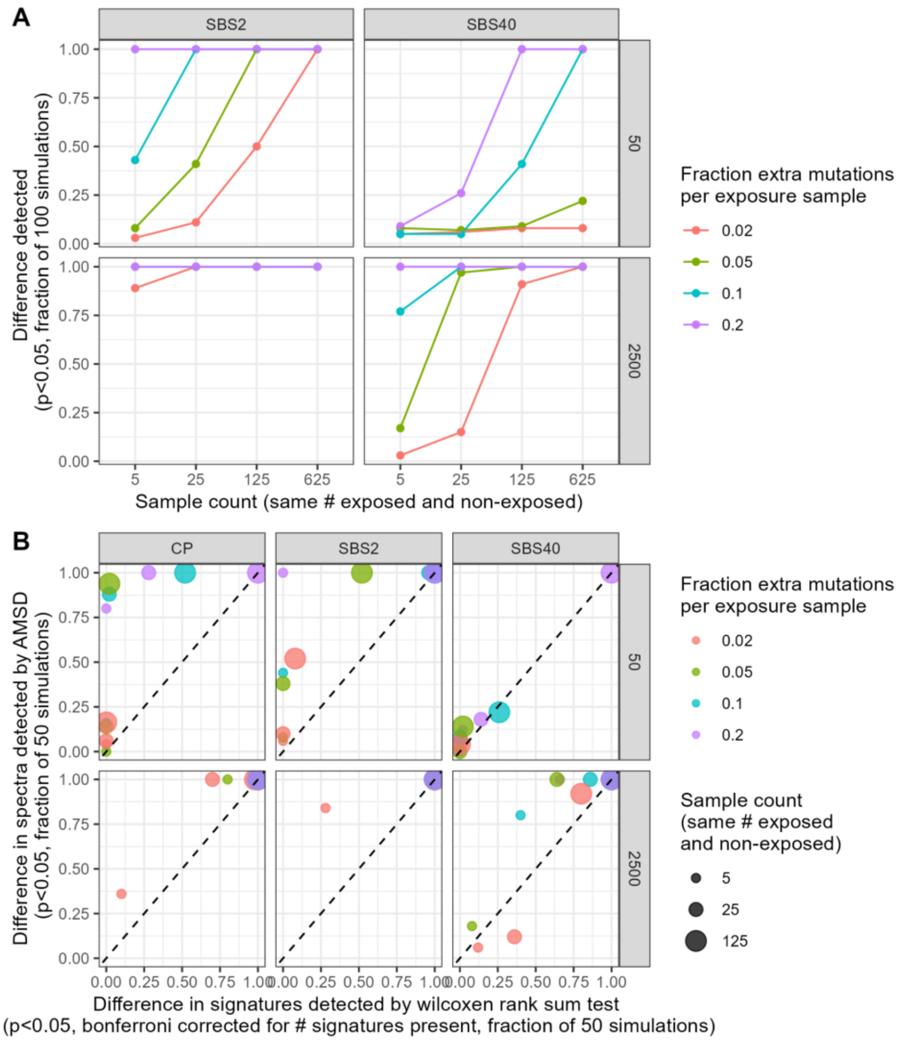

Supp Fig 1. AMSD's power of detection in simulated data sets

(**A**). The fraction of trials in which AMSD detects significant difference between two groups was detected given four variable parameters: variable signature present in one group ("spiky" SBS2 or "flat" SBS40: left and right columns), number of mutations per sample (2500 to represent whole genome sequencing, 50 to represent whole exome sequencing: top and bottom rows), number of samples per group (5- 625 to represent the range from a small experimental study like Riva et al. to a large observational study like TCGA: x-axes), and number of extra mutations per sample in the exposure group as a percentage of total mutations (2-20%: colors). (**B**). The fraction of trials in which a significant difference between two groups was detected by AMSD or by signature fitting. The preponderance of data points above the diagonal implies that AMSD is generally the more powerful approach, except in cases where both methods perform well. An additional spiky signature was added to test the robustness of these methods to the incompleteness of the COSMIC catalog: for this challenge, we chose the signature of exposure to CP = Cyclophosphomide, a chemotherapy that causes DNA damage with a signature that is not represented in COSMIC.

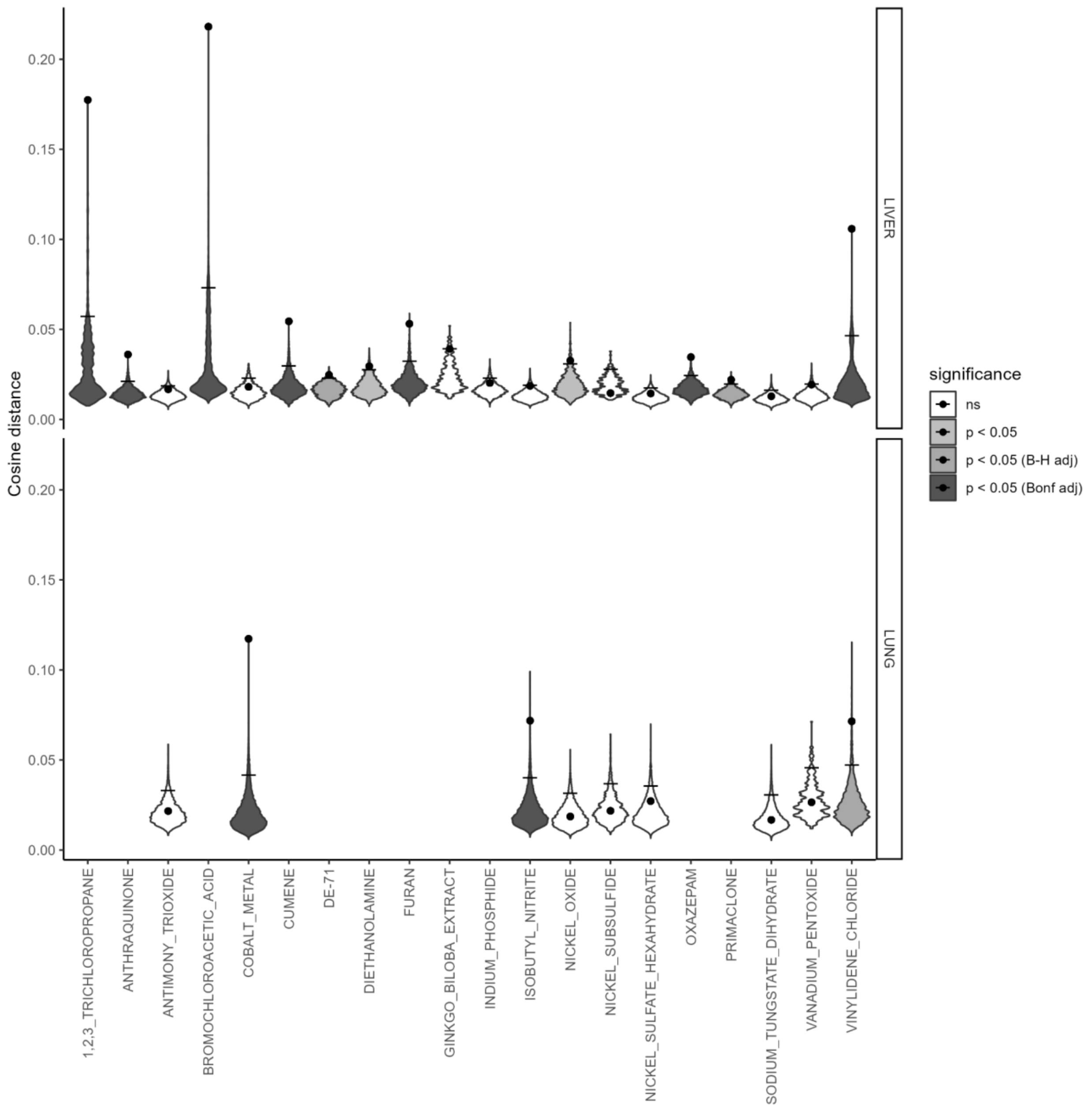

Supp Fig 2. AMSD results for all mouse carcinogen exposures

Violin plots for each AMSD comparison presented in Figure 3A. Violins display the null distribution of random samplings (100,000 each), points display the observed cosine distance between the carcinogen-exposed and spontaneous tumors, and lines represent the 95% quantile p=0.05 threshold. Violin plots are also colored by the corresponding significance threshold (unadjusted, Benjamini-Hichberg adjusted, or Bonferroni adjusted).

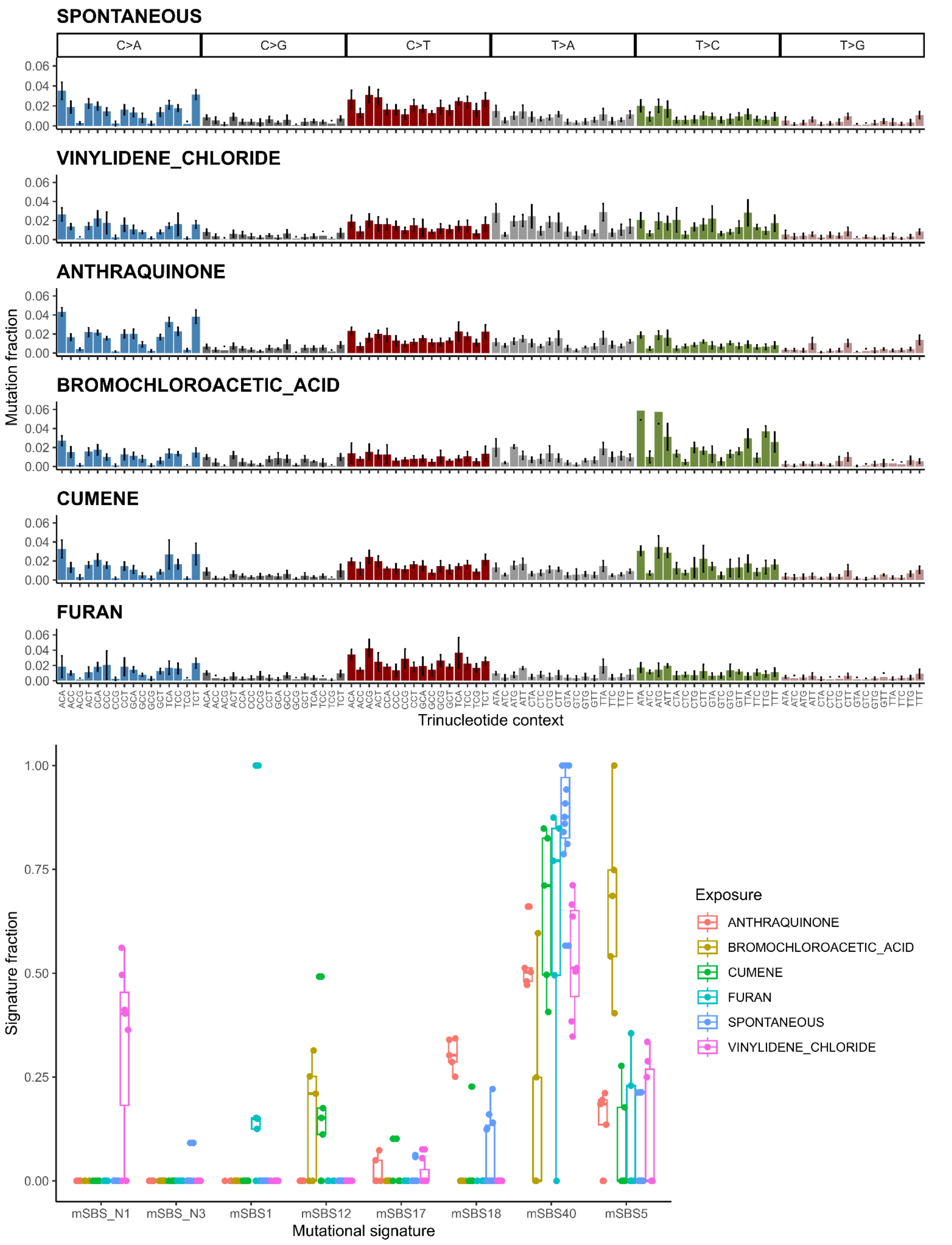

Supp Fig 3. Spectrum and signature results for other significant mouse liver carcinogens
Aggregate mean mutation spectra for mouse liver tumors, with standard deviation in error bars, for spontaneous tumors and carcinogen-exposed liver tumors that were significantly different after Bonferroni correction and not already included in Figure 3 (top). Signature exposures for each sample grouped by exposure and summarized with a boxplot (bottom).

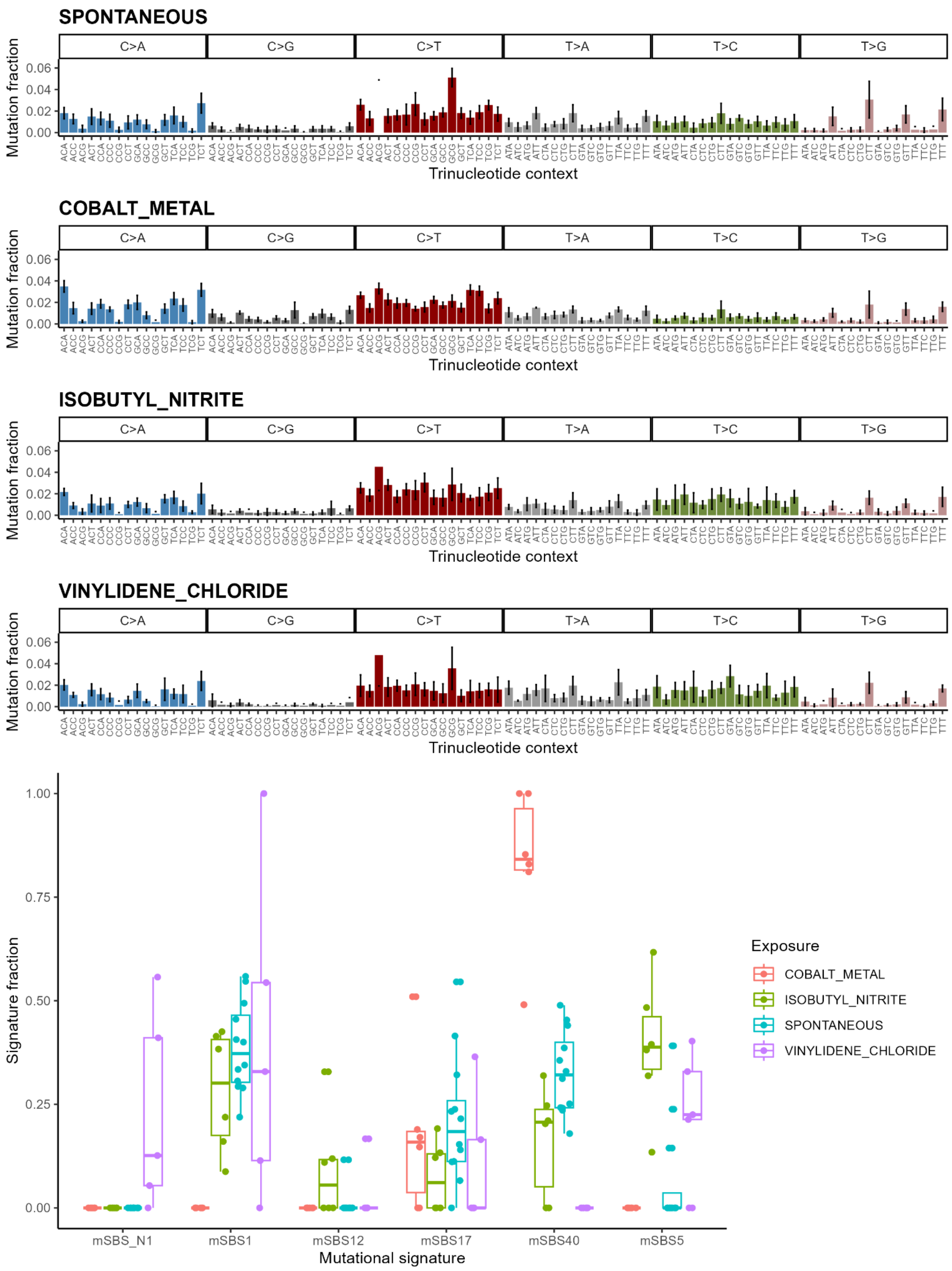

Supp Fig 4. Spectra and signature results for significant mouse lung carcinogens

Aggregate mean mutation spectra for mouse lung tumors, with standard deviation in error bars, for spontaneous tumors and carcinogen-exposed lung tumors that were significantly different after Benjamini-Hochberg correction (top). Signature exposures for each sample grouped by exposure and summarized with a boxplot (bottom).

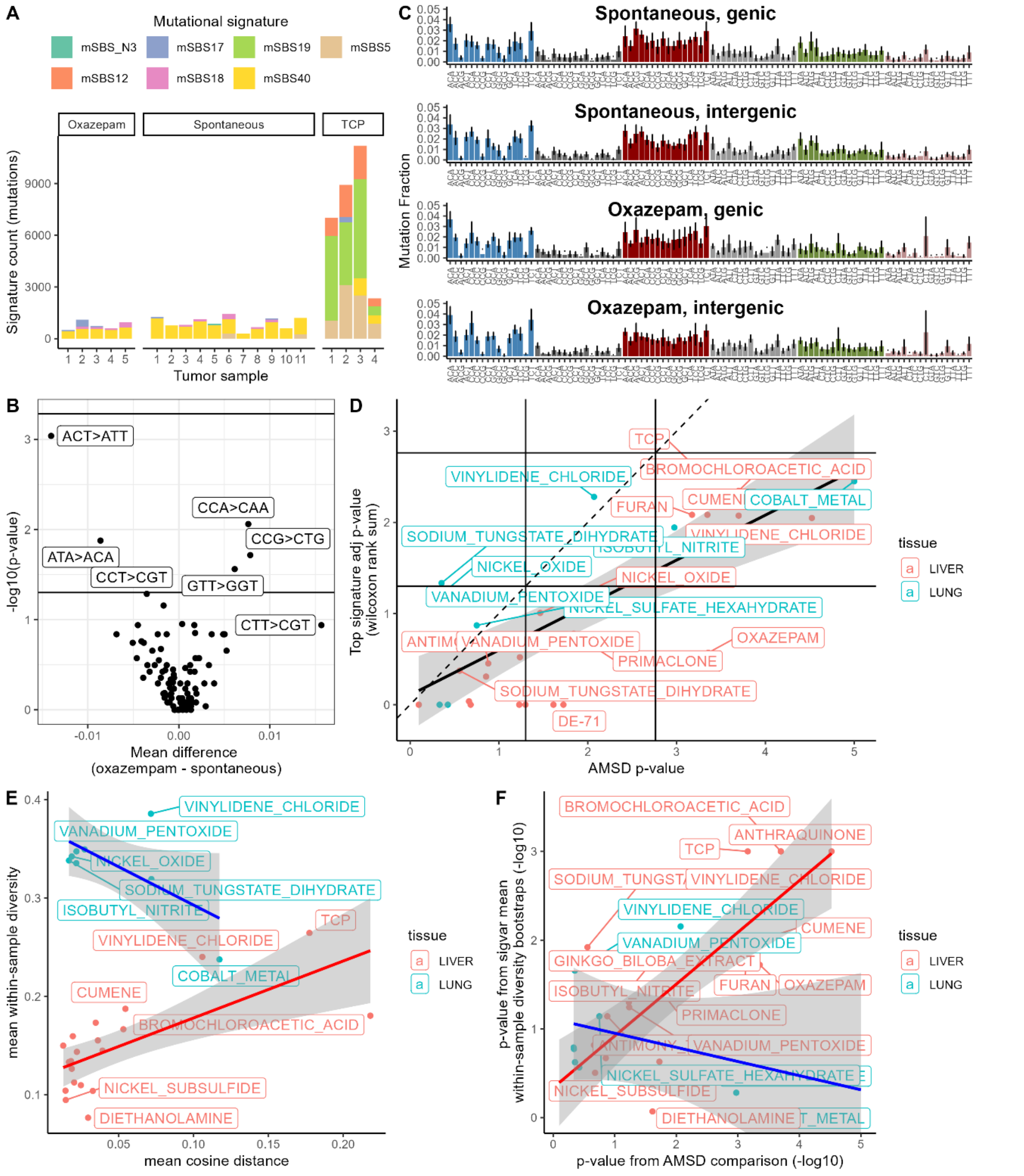

Supp Fig 5. AMSD results comparisons for mouse carcinogen analyses

(**A**) Stacked bar plots of absolute mutation counts of mutational signature exposures for each sample in Figure 3C. (**B**) Volcano plot of each trinucleotide mutation fraction compared by Wilcoxon rank sum between oxazepam-exposed liver tumors and spontaneously arising liver tumors. Solid horizontal lines represent significance thresholds of p=0.05 and bonferroni-corrected p=0.05/96. (**C**) Aggregate mean mutation spectra for spontaneous, TCP-exposed, and oxazepam-exposed liver tumors, with standard deviation in error bars, divided by whether mutations were genic or intergenic. (**D**) Comparison of *p*-values (-log10 scale) from AMSD or Wilcoxon rank sum-based signature method for mouse analyses. Dashed diagonal line represents 1:1 ratio, while solid diagonal lines represent linear regression by tumor type, with 95% confidence interval in grey. Solid vertical and horizontal lines represent significance thresholds of p=0.05 and bonferroni-corrected p=0.05/29. Comparisons of (**E**) aggregate mean spectrum cosine distance to mean within-sample diversity (metric from Morrison et al.), and (**F**) the *p*-value significance of spectrum difference by AMSD to bootstrapping significance of within-sample diversity. Each point in A/C/D represents an exposure versus spontaneous tumors of the same tumor type, liver (red) or lung (blue), from Riva et al. (*n* = 29).

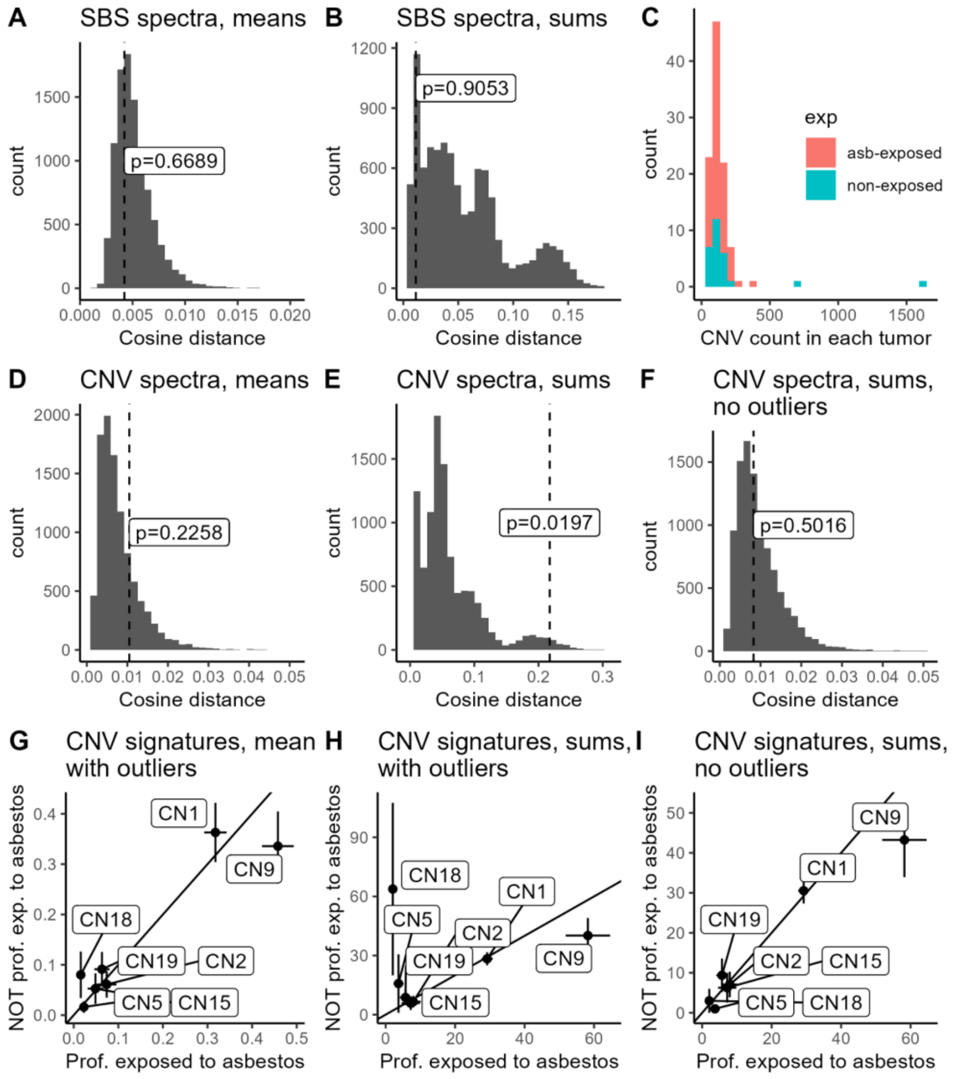

Supp Fig 6. Asbestos exposure influence on CNV spectra

AMSD results for SBS spectra (**A, B**) and CNV spectra (**D, E, F**), comparing mesothelioma spectra for patients professionally exposed to asbestos to those not professionally exposed to asbestos, either weighting all samples equally ("means"), or aggregating all mutations so that samples are weighted by mutation count ("sums"). Histograms show the null distribution expectations from 10,000 random samplings in relation to the real cosine distance between the aggregate spectra and corresponding *p*-value, marked with a dashed line. (**C**) Total CNV count per tumor, showing two high-CNV outliers that were not professionally exposed to asbestos. (**F**) Histogram of AMSD result for CNV sums after dropping >500 CNV outliers. Signature exposures (**G)** or counts (**H, I**) for each COSMIC v3.1 CN signature, comparing the average and standard error of the mean (error bars) by whether or not patients were professionally exposed to asbestos.

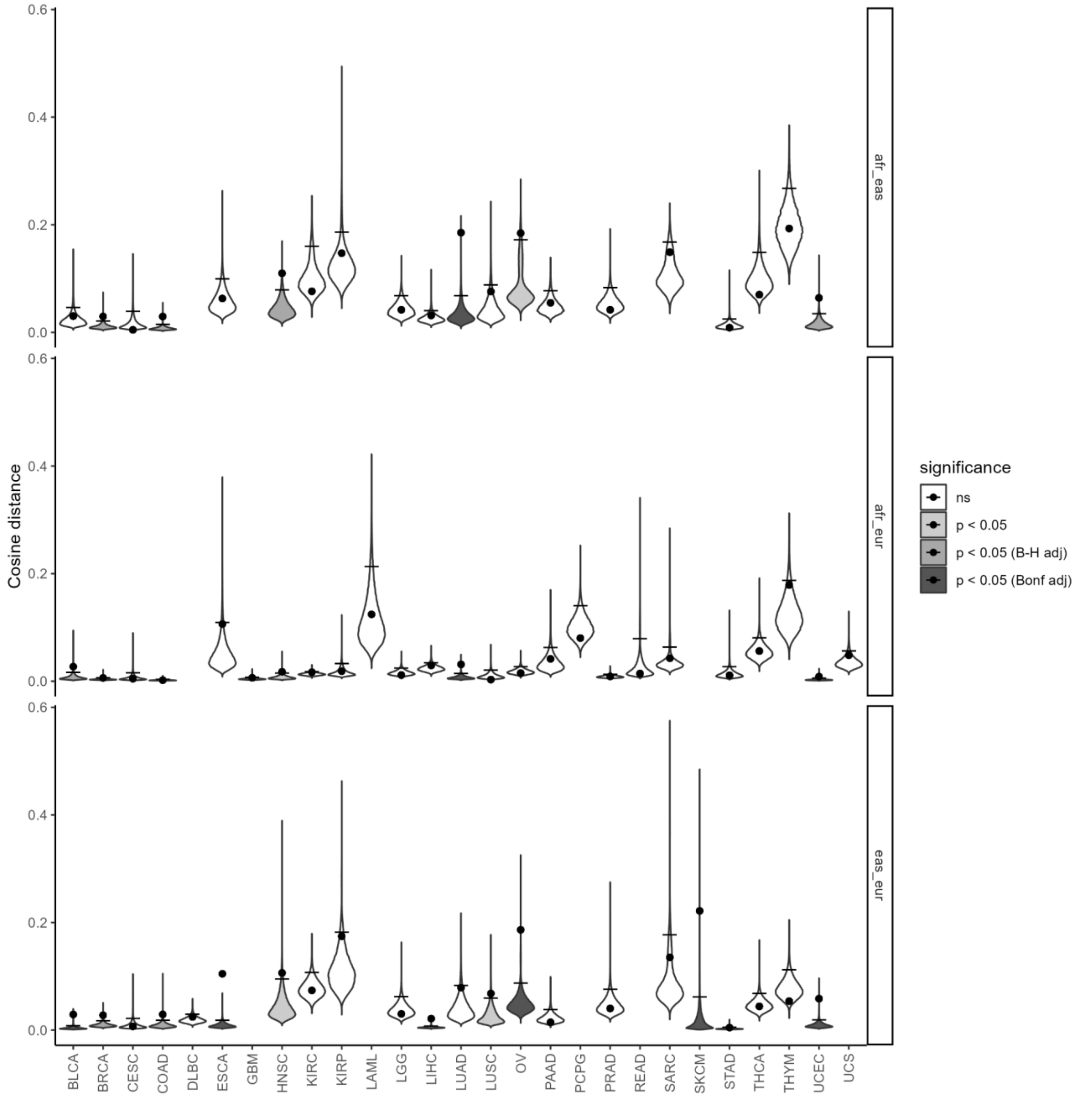

Supp Fig 7. AMSD results for all TCGA ancestry comparisons

Violin plots for each AMSD comparison presented in Figure 4A. Violins display the null distribution of random samplings (100,000 each), points display the observed cosine distance between the carcinogen-exposed and spontaneous tumors, and lines represent the 95% quantile p=0.05 threshold. Violin plots are also colored by the corresponding significance threshold (unadjusted, Benjamini-Hochberg adjusted, or Bonferroni adjusted).

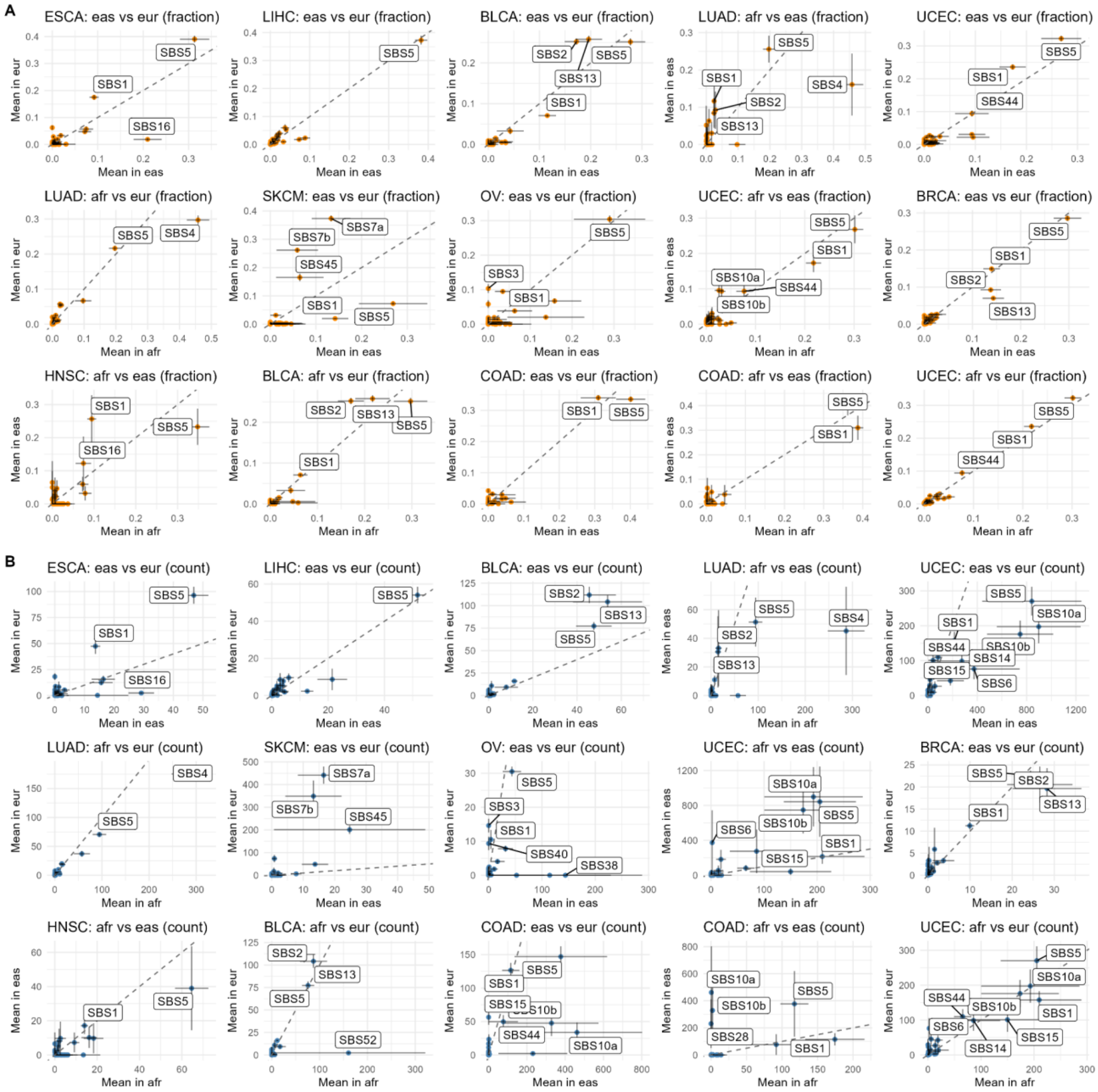

Supp Fig 8. Relative signature exposures in tumor pairs with significantly different mutation spectra ($p < 0.05$)
(**A**). Mean signature exposure fractions for each ancestry group for tumor types where ancestry-associated mutation spectra differ significantly ($p < 0.01$), with each dot denoting a COSMIC v3.4 SBS signature. (**B**).Same comparisons, but with the mean absolute signature exposure mutation counts rather than fraction (samples not weighted evenly). Diagonal line denotes 1:1 ratio. Error bars denote standard error of the mean. Choice of sample weighting makes the most impact when the mutation load is very different between ancestry groups, as in the case of UCEC, where tumors from East Asian patients are more likely to have a hypermutator phenotype.

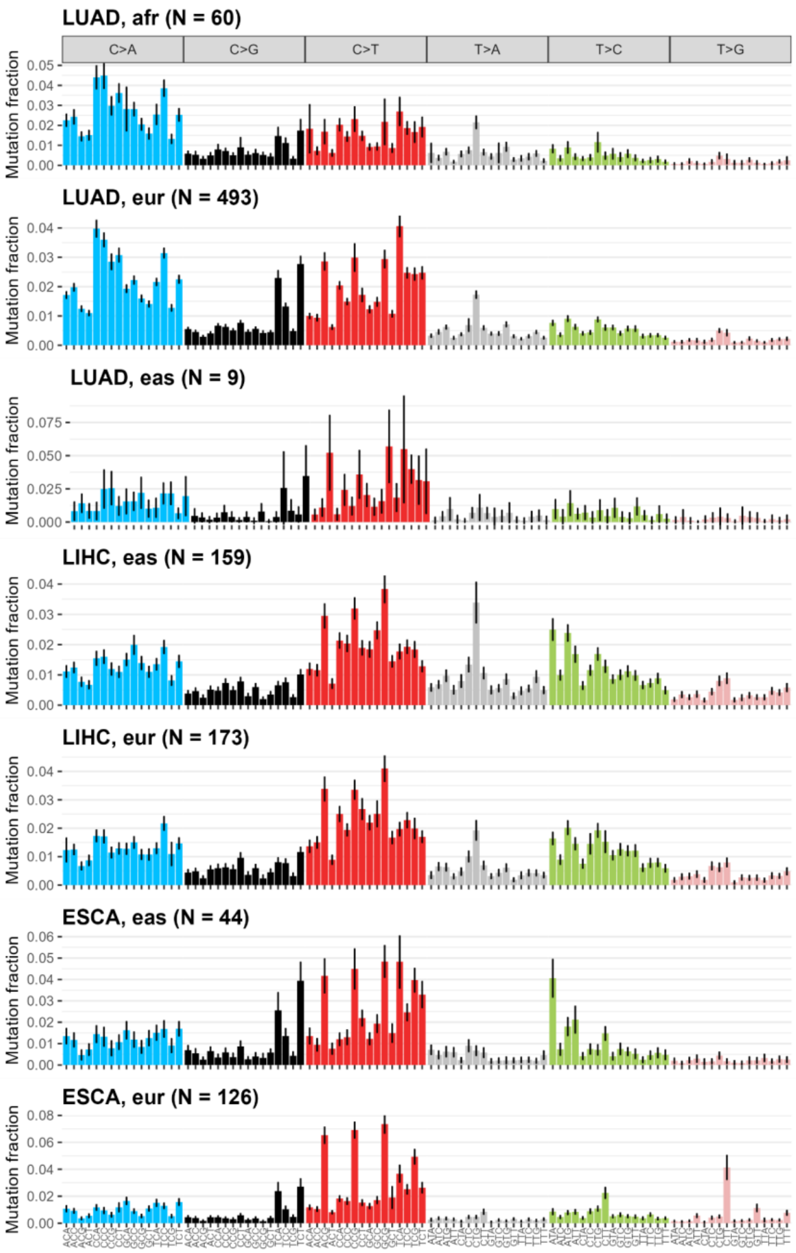

Supp Fig 9. Aggregate spectra for Lung adenocarcinoma (LUAD), liver hepatocellular carcinoma (LIHC), and esophageal carcinoma (ESCA) by ancestry. Bars denote spectra means (all samples weighted equally). Error bars denote 95% confidence intervals from the standard error of the mean ($\sigma / \sqrt{n}$). Notable differences include C>A in LUAD (SBS4), T>A and T>C in LIHC (SBS22 and SBS16, respectively), and T>C and T>G in ESCA (SBS16 and SB17b, respectively).

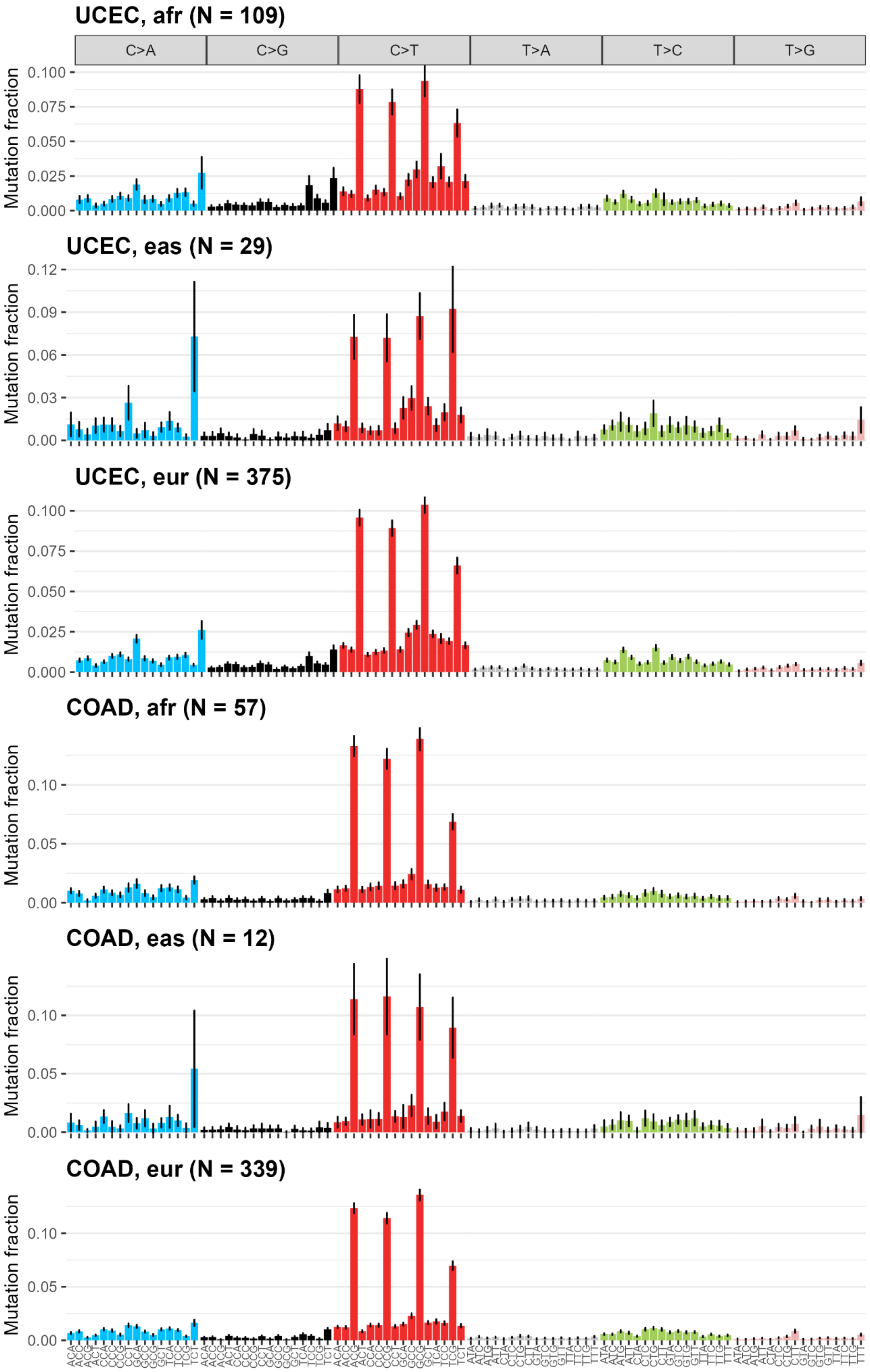

Supp Fig 10. Aggregate spectra results for Uterine (Uterine corpus endometrial carcinoma - UCEC) and colorectal adenocarcinoma (COAD) cancers. Bars denote spectra means (all samples weighted equally). Error bars denote 95% confidence intervals from the standard error of the mean ($\sigma / \sqrt{n}$)). Notable differences include TCT>TAT (SBS10a) and TCG>TTG (SBS10b).

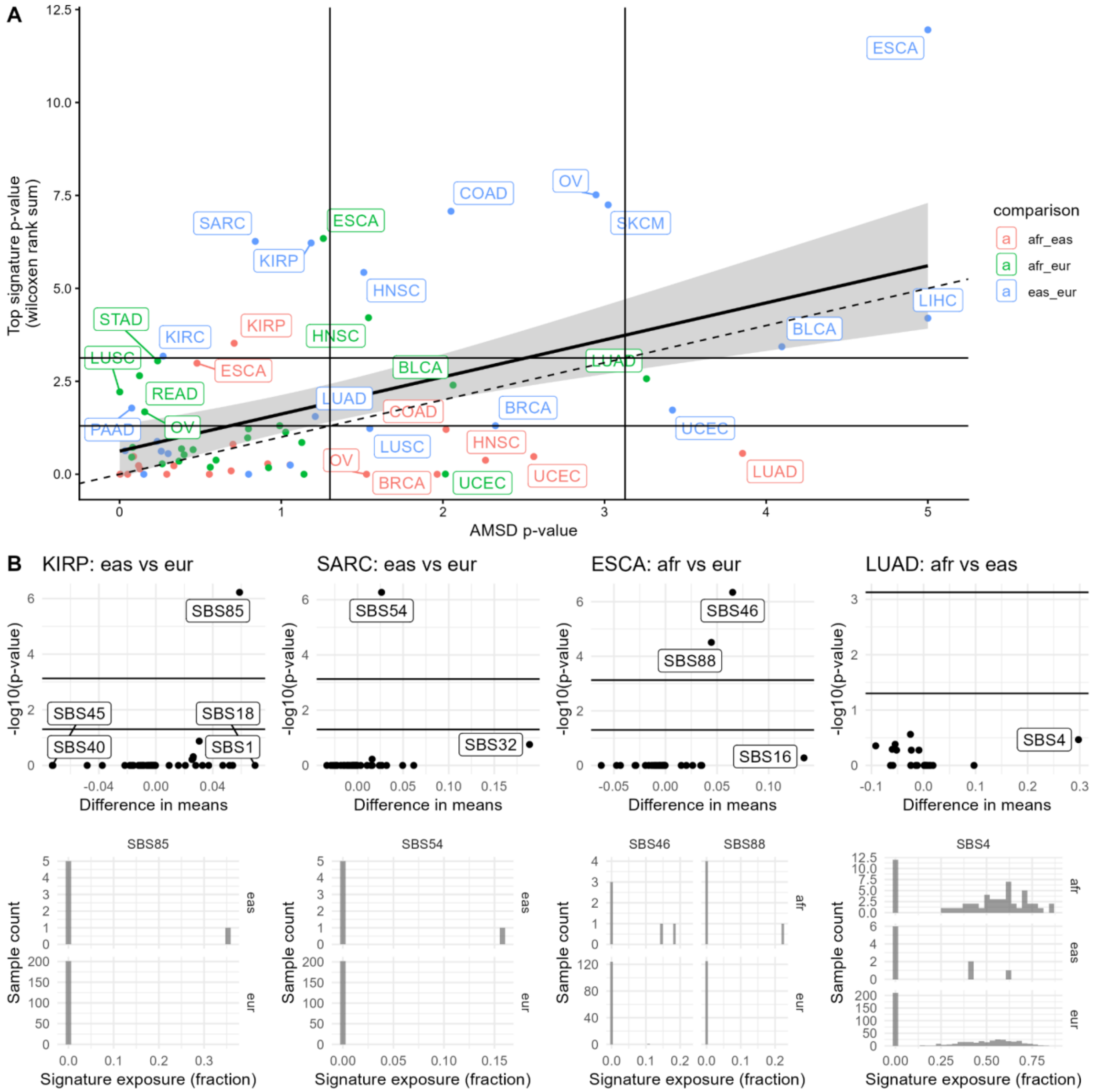

Supp Fig 11. AMSD results compared to signature fitting results for TCGA ancestry analyses

(**A**) Comparison of *p*-values (-log10 scale) from AMSD or Wilcoxon rank sum-based signature method for TCGA ancestry analyses. Dashed diagonal line represents 1:1 ratio, while solid diagonal lines represent linear regression by tumor type, with 95% confidence interval in grey. Solid vertical and horizontal lines represent significance thresholds of p=0.05 and bonferroni-corrected p=0.05/67.
(**B**) Data behind signature significance drivers for the largest outliers that were detected by signatures but not AMSD (KIRP, SARC, ESCA) or by AMSD but not signatures (LUAD). Upper plots are volcano plots for each Wilcoxon rank sum comparison, with each point representing a COSMIC v3.4 signature, while lower plots are histograms of signature exposure fractions for most significant signature(s), separated by ancestry.

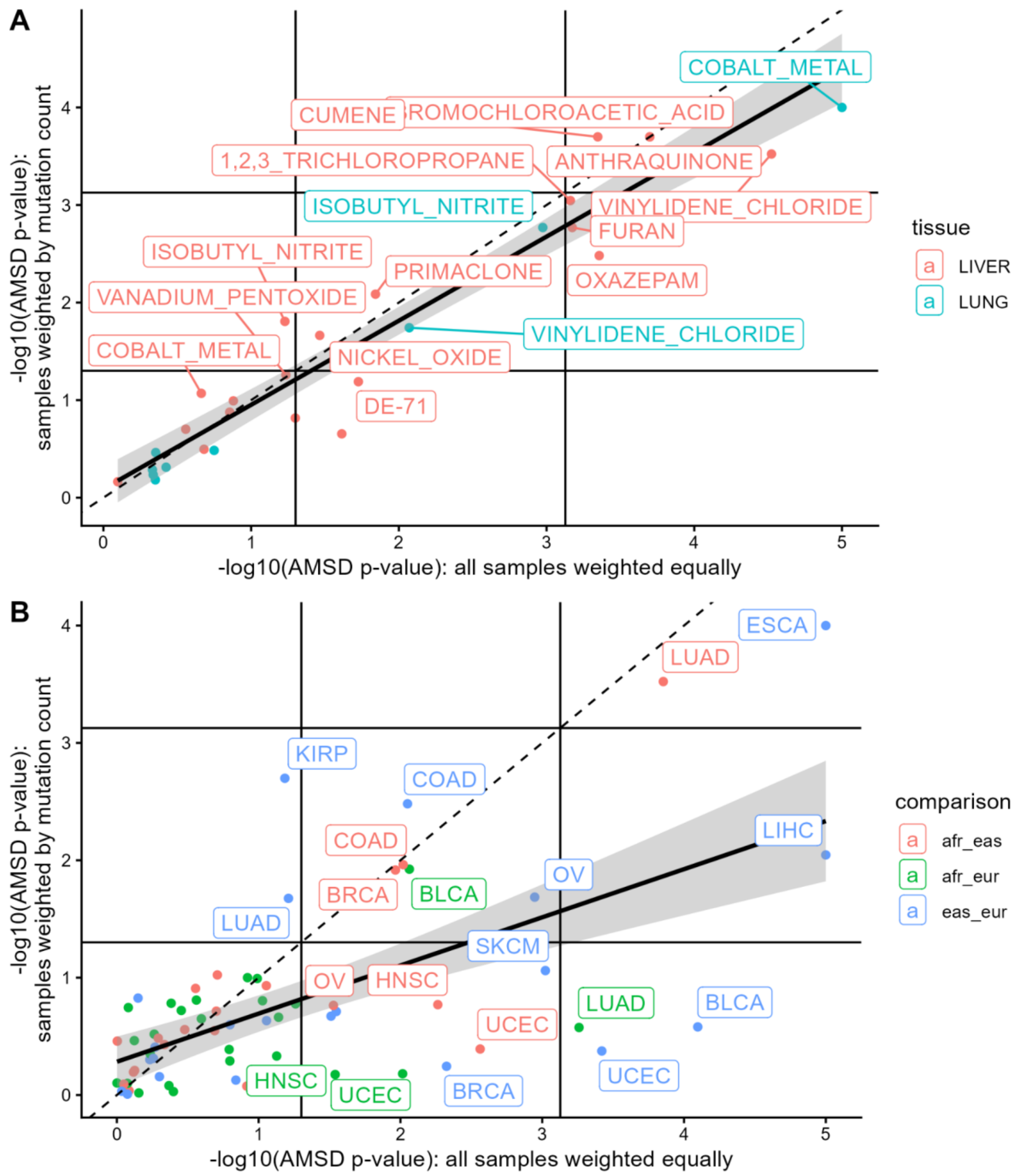

Supp Fig 12. AMSD results when run with all samples weighted equally (default) versus samples weighted by mutation count

Comparison of *p*-values from AMSD when all samples are weighted equally (default: "mean" method) versus when samples are weighted by mutation count ("sum" method) for mouse (**A**) and TCGA ancestry (**B**) analyses. Dashed diagonal line represents 1:1 ratio, while solid diagonal lines represent linear regression by tumor type, with 95% confidence interval in grey. Solid vertical and horizontal lines represent significance thresholds of p=0.05 and bonferroni-corrected p=0.05/29 or p=0.05/67.
